# Awake Fiberoptic Nasotracheal Intubation and Anesthetic Management of a Patient With a Compressed and Deviated Airway From a Massive Thyroid Goiter: A Case Report

**DOI:** 10.7759/cureus.35278

**Published:** 2023-02-21

**Authors:** Didem Tan, Xuechao Zhang

**Affiliations:** 1 Anesthesiology, Hackensack University Medical Center, Hackensack, USA

**Keywords:** tracheal narrowing, tracheal compression, difficult airway management, naso endotracheal intubation, awake fiberoptic intubation, difficult airway intubation

## Abstract

Difficult airway management is a challenge for anesthesiologists, requiring proper assessment, planning, and sometimes a multidisciplinary approach to establish a secure airway. Here we present a case where the patient had a large thyroid goiter with significant tracheal compression. Due to the large size of the thyroid mass and the location of tracheal narrowing, fiberoptic intubation appeared to be challenging, and a surgical airway was not a viable option to obtain a secure airway for a total thyroidectomy. This case report discusses awake fiberoptic intubation and intraoperative anesthetic management of a patient with known airway compression and explores the alternative method for obtaining a definitive airway through venovenous extracorporeal membrane oxygenation.

## Introduction

Tracheal compression and displacement due to an enlarged thyroid mass is an example of a difficult airway requiring a detailed plan for airway and anesthetic management. The current difficult airway algorithm does not provide clear guidelines for scenarios where surgical airways, such as cricothyrotomy or tracheostomy, are not feasible [1]. Awake fiberoptic intubation is the recommended method for securing difficult airways, but it is still associated with a high risk depending on the location of tracheal narrowing and the extent of airway compression [1]. Here we present a case where the patient had a massive thyroid goiter with resulting severe proximal tracheal compression. The patient was successfully intubated via awake fiberoptic nasotracheal intubation. In this case report, we present the challenges of intraoperative mechanical ventilation in a patient with a compressed airway. We also discuss the utility of venovenous extracorporeal membrane oxygenation (VV ECMO) in case all other conventional airway management methods were to fail.

## Case presentation

**Figure 1:**
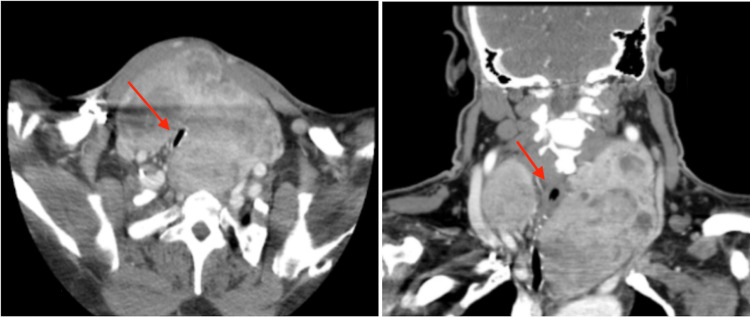
A CT scan of the patient’s neck in axial view (left) and coronal view (right). A CT scan of the neck was obtained with contrast. The compressed and deviated airway is shown by the red arrows.

A 55-year-old male with a past medical history of hypertension, thyroid goiter, and a prior tracheostomy after a motor vehicle accident with subsequent decannulation presented with progressive shortness of breath and hemoptysis. The patient had a diagnosis of thyroid goiter for six years but was lost to follow-up. When the patient initially presented to an outside hospital emergency room for breathing difficulties, a computerized tomography (CT) scan of the neck was obtained. It showed an enlarged thyroid gland with superior mediastinal extension, the left lobe measuring 12.8 x 7.7 x 6.1 cm and the right lobe measuring 9.1 x 9.2 x 4.6 cm (Figure 1). As per the radiology report, the trachea was compressed and displaced to the right, measuring approximately 3 mm in transverse diameter at its narrowest point (Figure 1). There was no major vascular obstruction noted in the report. The patient had normal thyroid gland function, with free thyroxine (T4), free triiodothyronine (T3), and thyroid stimulating hormone (TSH) levels all within normal limits. The patient was taken to the operating room for a total thyroidectomy; however, fiberoptic nasotracheal intubation attempts by both anesthesiologists and otolaryngologists (ENT) were unsuccessful due to the observed severe airway compression immediately below the vocal cords. The patient was then transferred to our institution for further evaluation and management.

**Figure 2:**
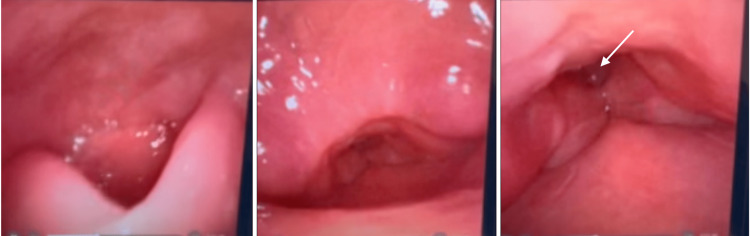
A preoperative exam of the patient's larynx by flexible laryngoscopy. The images show laryngeal edema and inflammation. The vocal cords are indicated by the white arrow.

In our institution, the ENT team performed a bedside fiberoptic laryngoscopy that showed extensive laryngeal edema and inflammation with a narrow glottic opening where the vocal cords were difficult to visualize (Figure 2). The patient displayed mild respiratory discomfort without any significant stridor or dyspnea at rest. However, multiple episodes of apnea and oxygen desaturation were observed during his sleep, with saturation of peripheral oxygen (SpO2) readings at a low 80%. The patient did not have a prior sleep study for the diagnosis of obstructive sleep apnea (OSA), but a STOP-Bang score of six indicated a high risk of having moderate to severe OSA. His predicted obstructive sleep apnea further presented a challenge in the perioperative airway management along with his airway compression. A thorough discussion of the risks and benefits of multiple airway management strategies for total thyroidectomy was conducted among the patient, ENT, and anesthesiology teams. Given the fact that the patient was not in immediate respiratory distress when he was awake and at rest, it was decided to make another attempt at awake fiberoptic nasotracheal intubation in the operating room. If unsuccessful, the plan was to proceed with cannulation for VV ECMO prior to total thyroidectomy.

**Figure 3:**
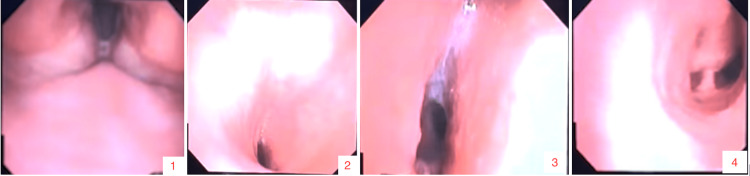
Awake fiberoptic intubation of the patient with flexible bronchoscopy. Pictures are numbered and labeled in the right lower corner. Image 1 shows the larynx with edematous arytenoids and vocal cords. Images 2 and 3 show the compressed airway. The carina is visualized in image 4.

The patient was brought into the operating room after receiving nebulized lidocaine treatment and a scheduled dose of dexamethasone. He was placed in a sitting position on the operating table. Standard American Society of Anesthesiologists (ASA) monitors were placed. His oxygen saturation (SpO2) was 98% on room air. The patient’s left naris was anesthetized with topical lidocaine gel and serially dilated with nasal trumpets in sizes 26, 30, and 32. The patient was then given a lidocaine 4% solution to gargle to anesthetize the posterior pharynx and larynx. Transtracheal and superior laryngeal nerve injections were not possible. The patient was not given any sedatives to maintain spontaneous respirations. A 4.0 mm pediatric flexible fiberoptic bronchoscope was loaded with a size 5.5 endotracheal microlaryngeal tube (MLT) and then slowly introduced into the left naris. The larynx was visualized, and then the bronchoscope was advanced into the trachea, where significant narrowing was again noted (Figure 3). The tracheal deviation due to the mass effect was also appreciated. The scope was able to pass through the most compressed area, and the carina was visualized (Figure 3). However, the 5.5 MLT was not able to pass. The patient’s SpO2 dropped to 88% at its lowest during the first attempt but quickly recovered with supplemental oxygenation through a non-rebreather mask. At the second attempt, a size 5.0 MLT was successfully advanced past the compressed area. After confirming the placement of the MLT with the visualization of carina by bronchoscope and end-tidal CO2, the patient was administered fentanyl, propofol, and rocuronium intravenously. The MLT was secured to the nasal septum with silk sutures.

Intraoperatively, high peak airway pressures of 40-50 cmH2O were encountered with pressure-limited and lower than preset tidal volumes. The small caliber of the endotracheal tube (ETT), the extension of the neck for surgical exposure, and the surgeon’s manipulation of the thyroid mass were suspected to be the causes of the difficulties in achieving optimal ventilation, which rendered the inhalational anesthetics less effective. Therefore, a continuous total intravenous anesthesia (TIVA) strategy with dexmedetomidine and remifentanil was utilized. Rocuronium was used to keep the patient paralyzed to facilitate respiratory mechanics.

**Figure 4:**
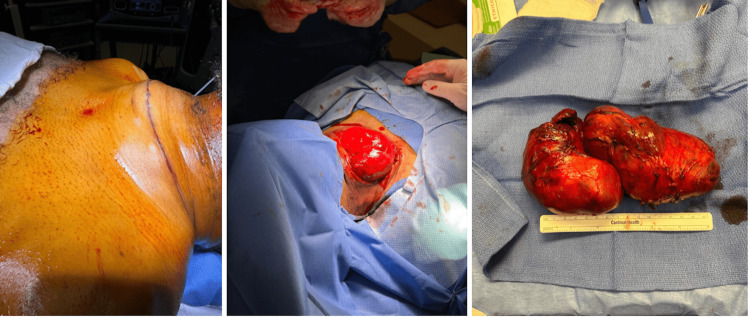
Images of the thyroid mass before (left), during (middle), and after (right) surgical removal.

The surgical removal of the thyroid mass corrected the tracheal deviation secondary to the mass effect (Figure 4). As a result, the previously suture-secured ETT migrated to the right main stem. Under fiberoptic bronchoscopy guidance, the position of the ETT was readjusted and the airway was re-examined. At the completion of the surgery, the muscle relaxation was fully reversed with sugammadex. The patient’s respiratory mechanics returned to normal with the removal of the mass. He achieved adequate tidal volume and normal peak airway pressure under spontaneous breathing.

Unlike traditional total thyroidectomy procedures with nerve monitoring, the use of a neuro integrity monitoring (NIM) tube was not an option in this case. Therefore, there was a concern for possible vocal cord immobility due to the high risk of recurrent laryngeal nerve injury during the surgery. Prior to extubation, the patient’s airway was re-examined with a video laryngoscope, which showed bilateral mobile vocal cords without airway edema or bleeding. The ENT surgeon was on standby during the extubation period. The patient was successfully extubated and then transferred to the intensive care unit for further observation and recovery. The patient reported marked improvement in respiration and phonation postoperatively.

## Discussion

Anticipated difficult airways are a major concern for anesthesiologists. It requires an extensive evaluation of the patient and the formulation of a strategy by both the surgical and anesthesiology teams. For patients who have known tracheal narrowing due to mass compression, induction of general anesthesia may cause total airway obstruction and make it impossible to establish a secure airway [2]. The difficult airway algorithm provides guidelines for unanticipated difficult airways, but there is no clear algorithm for anticipated difficult airways where invasive airway management is not possible [1]. If an invasive airway approach is not feasible after failed awake intubation attempts, the use of ECMO is the only viable option for respiratory support in anesthetic management [1-4]. There are two forms of ECMO: veno-arterial (VA) ECMO, which provides complete respiratory and cardiac support, and venovenous (VV) ECMO, which bypasses only the respiratory system [2-4]. For difficult airway cases, VV ECMO can be used before the induction of general anesthesia to prevent airway complications [2-4]. The cannulation for ECMO can be preemptively performed either in the operating room or intensive care unit under local anesthesia [4,5]. TIVA is the mode of anesthesia for patients requiring VV ECMO due to the unpredictable effectiveness of inhaled anesthetic delivery with low tidal volumes and increased dead space [5,6].

In our case, the ENT and anesthesiology teams decided on awake nasotracheal intubation as the patient was not in immediate respiratory distress and a highly experienced and skilled surgeon was confident in establishing a secure airway fiberoptically. A cardiac surgeon and a perfusionist were available in case of a need for urgent VV ECMO cannulation. The patient was treated with corticosteroids prior to the surgery in an effort to decrease the mass size and laryngeal edema. The nasotracheal intubation pathway was anesthetized with nebulized lidocaine treatment, liquid lidocaine gargle, and topical lidocaine gel in the nostrils, as well as on the intubation equipment, to make the intubation process more tolerable for the patient. We also utilized the spray-as-you-go technique during intubation for effective topical anesthesia of the airway [7].

The management of respiratory mechanics for adequate ventilation was another challenging part of the case. Low tidal volumes and high peak airway pressures are commonly encountered in cases of airway compression and the use of ETT in small diameters. Inadequate ventilation of the lungs limits the uptake and effectiveness of inhaled anesthetics [8]. Intravenous anesthetics are useful either as the primary or adjunct mode of anesthesia [9]. They provide a fast onset of action and rapid recovery [9]. We used remifentanil for its analgesic effect and its reliable, short context-sensitive half-life, allowing a rapid emergence without prolonged respiratory depression [10]. We also used dexmedetomidine because, in addition to its sedative and analgesic effects, it does not cause respiratory depression, which led to smooth extubation in our case [10].

Tracheomalacia is a complication that can occur following thyroidectomy in patients with a prolonged large goiter [11]. The airway compression caused by the thyroid mass can damage the tracheal wall and may lead to airway collapse during extubation [11]. If the patient is suspected to have severe tracheomalacia, keeping the patient intubated following thyroidectomy would be the safest option to keep the airway secured [11]. In our case, the surgeon's exam of the trachea did not show a major concern for tracheomalacia. After the thyroidectomy, the extrinsic compression of the airway resolved and the respiratory mechanics of ventilation normalized. The patient demonstrated adequate tidal volume on spontaneous breathing and was able to follow commands. The decision for a trial of extubation was made in discussion with both the ENT and anesthesiology teams. The patient was safely extubated, and his airway remained patent post-extubation.

## Conclusions

Overall, airway and anesthetic management of a patient with a known or anticipated difficult airway requires detailed assessment and planning. Anesthesiologists need to consider multiple factors for a successful operative course, such as preoperative preparations, the selection of anesthetic techniques, and alternative methods for establishing a secure airway. When all other conventional methods fail to obtain a definitive airway, the last option is the utilization of VV ECMO.
